# Pharmacokinetic study on the interaction between succinic acid and irbesartan in rats and its potential mechanism

**DOI:** 10.1080/13880209.2021.2002370

**Published:** 2021-11-24

**Authors:** Yongpeng Wang, Ruping Rui, Xiaoyan Zhang, Bin Sun

**Affiliations:** a Department of Cardiovascular Medicine, Yidu Central Hospital of Weifang, Weifang, Shandong, China; b Qingzhou Tanfang Town Central Health Center, Weifang, Shandong, China; c Department of Emergency, Yidu Central Hospital of Weifang, Weifang, Shandong, China

**Keywords:** Cardiovascular disease, drug–drug interaction, CYP2C9, amber

## Abstract

**Context:**

Succinic acid and irbesartan are commonly used drugs in cardiovascular disease treatment. The interaction might occur during their co-administration, which was still unclear.

**Objective:**

To reveal the effect of succinic acid on the metabolism of irbesartan and its potential mechanism.

**Materials and methods:**

The Sprague-Dawley rats (*n* = 6) were treated with a single dose of 30 mg/kg irbesartan (control) or the co-administration with the pre-treatment of 200 mg/kg succinic acid for 7 d. The effect of succinic acid on the metabolic stability and the activity of CYP2C9 was evaluated in rat liver microsomes.

**Results:**

Succinic acid increased the AUC (5328.71 ± 959.31 μg/L × h vs. 3340.23 ± 737.75 μg/L × h) and prolonged the half-life of irbesartan (from 12.79 ± 0.73 h to 20.59 ± 6.35 h). The *T*_max_ (2.83 ± 0.75 h vs. 3.83 ± 1.10 h) and clearance rate (3.46 ± 1.13 L/h/kg vs. 6.91 ± 1.65 L/h/kg) of irbesartan was reduced by succinic acid. Consistently, succinic acid improved the metabolic stability (half-life from 23.32 ± 3.46 to 27.35 ± 2.15 min, intrinsic clearance rate from 59.43 ± 6.12 to 50.68 ± 5.64 μL/min/mg protein). Succinic acid was also found to inhibit the activity of CYP2C9 with the IC_50_ value of 13.87 μM.

**Discussion and conclusions:**

Succinic acid increased the system exposure of irbesartan via inhibiting CYP2C9. The experiment design of this study also provides a reference for the further validation of this interaction in humans.

## Introduction

The combination of diverse drugs or herbs is a common therapeutic strategy in the treatment of cardiovascular disease, especially in the use of traditional Chinese medicine (Parvez and Rishi 2019). The co-administration of different drugs could offer potential advantages, such as increasing efficacy and improving patient compliance, but it also brings adverse effects, such as therapy failure and toxicity (Rekić et al. 2017). Succinic acid is a major extraction of amber, which is commonly used in the therapy of arrhythmia. It has also been reported that succinic acid has various pharmacological effects, such as cardioprotective, antithrombotic, anti-inflammatory, and antibacterial (Tang et al. 2013; Zhang et al. 2014; Nissen et al. 2019). In the previous study, succinic acid has been demonstrated to inhibit the activity of cytochrome P450 enzymes (CYP450s), which are a series of enzymes responsible for the metabolism of a wide range of endogenous compounds (Wang et al. 2020). The activity of CYP450s has been considered as a critical factor that mediates drug-drug interaction during drug co-administration. Therefore, the inhibitory effect of succinic acid implies its potential interaction with other combined drugs.

Irbesartan is an angiotensin III receptor antagonist, which is applied in the clinical treatment of hypertension, metabolic syndrome, and cardiac disease (Markham et al. 2000; Vignier et al. 2014). CYPC2C9 was reported to be involved in the biotransformation of irbesartan, and the interactions of irbesartan with other drugs metabolized by CYP2C9 or affect the activity of CYP2C9 have been widely reported (Bourrie et al. 1999; Marino and Vachharajani 2001; Yang et al. 2016). The similar indications of succinic acid and irbesartan make them possible to appear in the same medical treatment. The interaction between these two drugs is of great importance, which might result in adverse effects on the therapeutic efficacy.

The co-administration of succinic acid and irbesartan was investigated *in vivo* and *in vitro* in the present study, to disclose the effect of succinic acid on the pharmacokinetics of irbesartan and provide a theoretical basis for the clinical combination of these two drugs.

## Materials and methods

### Animals

The ethical approval has been obtained from the Animal Care and Use Committee of Weifang Yidu Central Hospital. Adult male Sprague-Dawley rats (weighted 250–300 g) were obtained from Harlan Laboratories (Indianapolis, IN, USA). All animals had free access to food and water and were housed in a temperature and humidity-controlled environment. Before the experiment, all rats fasted with free access only to water for 12 h.

### Pharmacokinetic study

The pharmacokinetic profile of irbesartan was studied with oral administration of 30 mg/kg irbesartan. The rats were randomly divided into two groups with six rats of each including an irbesartan group and an irbesartan + succinic acid group. In the co-administrated group, the rats were pre-treated with 200 mg/kg succinic acid before the administration of irbesartan. The concentrations of irbesartan and succinic acid were selected according to previous studies (Jakobsdottir et al. 2013; Hedaya and Helmy 2015). After 0, 0.083, 0.25, 0.5, 1, 2, 3, 4, 6, 8, 12, and 24 h of the irbesartan administration, the plasma samples were collected and analyzed with LC-MS/MS.

### LC-MS/MS condition

The plasma concentration of irbesartan was analyzed with the help of Agilent 1290 series liquid chromatography and the 6460 triple-quadrupole mass spectrometer according to previous studies (Elgawish et al. 2019; Zhou et al. 2021). In the mass scan mode, the ion transitions of irbesartan and internal standard were *m/z* 429.1 → *m/z* 206.9 and *m/z* 256.2 → *m/z* 167.0, respectively.

### Metabolic stability study in rat liver microsomes

The effect of succinic acid on the metabolic stability of irbesartan was evaluated in rat liver microsomes. A pre-incubation of 5 min was performed before the experiments with 10 nM G-6-P, 1 mM NADP^+^, 4 mM magnesium chloride, 1 unit/mL of G-6-PDH, 30 μL rat liver microsomes, 100 μM irbesartan, and 0.1 M PBS buffer solution, and then irbesartan was added. For the succinic acid + irbesartan group, succinic acid was added and preincubating for 3 min followed by the addition of irbesartan. At 0, 1, 3, 5, 15, 30, and 60 min of the incubation, ice-cold acetonitrile was added to terminate the reaction and the mixture was collected for the following analyses.

The metabolic stability of irbesartan was assessed by the value of *in vitro* half-life calculated by the following equations:

$$t_{\frac{1}{2}}=\frac{0.693}{k};$$

$$\text{Intrinsic clearance }(\mathrm{Clint})\;(\mu L/\min/\text{mg protein})=v\times 0.693/t_{1/2}$$

V(μL/mg)=volume of incubation (μL)/protein in the incubation

### Effect of succinic acid on the activity of CYP2C9

The effect of succinic acid on the activity of CYP2C9 was estimated in rat liver microsomes according to the previous study (Wang et al. 2020). The incubation contained sodium phosphate buffer, magnesium chloride, diclofenac, and succinic acid with the dosage of 0, 2.5, 5, 10, 25, 50, and 100 μM. The incubation condition was similar to the above description, and after incubation, the mixture was centrifuged for the following analyses. The activity of CYP2C9 was evaluated by the concentration of metabolites with the help of HPLC.

### Statistical analysis

All data were represented as mean value ± SD obtained from triplicate repeated experiments. The corresponding pharmacokinetic parameters were calculated by the DAS 3.0 pharmacokinetic software (Chinese Pharmacological Association, China). The difference between groups was evaluated by Student’s *t*-test by SPSS 20.0 (Chicago, IL, USA). The difference was considered to be statistically significant when *p* < 0.05.

## Results

### Succinic acid increased the system exposure of irbesartan

**Figure 1.**
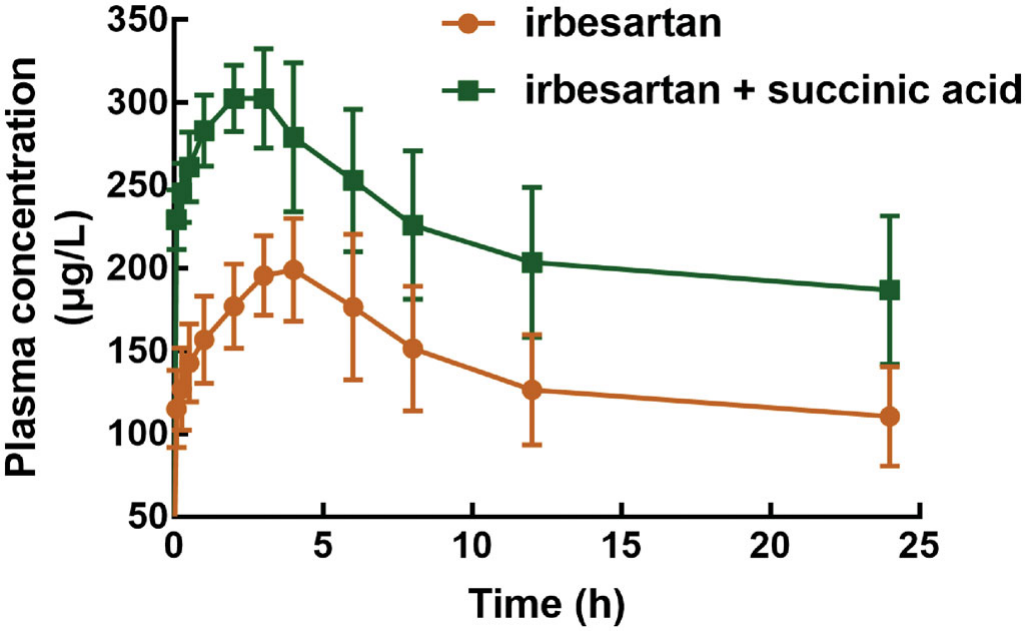
The plasma concentration-time curve of irbesartan (30 mg/kg) in the presence or absence of succinic acid (200 mg/kg).

The pharmacokinetic profile of irbesartan was significantly changed in the presence of succinic acid (Figure 1). The pharmacokinetic parameters of irbesartan in the presence of succinic acid were summarized in Table 1. Succinic acid improved the area under the curve (AUC) of irbesartan from 3340.23 ± 737.75 μg/L × h to 5328.71 ± 959.31 μg/L × h. The maximum concentration (*C*_max_) of irbesartan increased from 210.33 ± 26.08 μg/L to 315.3 ± 24.35 μg/L. Additionally, the half-life (*t*_1/2_) was prolonged from 12.79 ± 0.73 h to 20.59 ± 6.35 h. The *T*_max_ (2.83 ± 0.75 h vs. 3.83 ± 1.10 h) and clearance rate (3.46 ± 1.13 L/h/kg vs. 6.91 ± 1.65 L/h/kg) were reduced by succinic acid. All these results indicated that succinic acid increased the system exposure of irbesartan.

**Table 1.** Pharmacokinetic parameters of 30 mg/kg irbesartan in the presence or absence of 200 mg/kg succinic acid.

	Irbesartan	Irbesartan + succinic acid
AUC (0–*t*) (μg/L*h)	3340.23 ± 737.75	5328.71 ± 959.31***
*t*_1/2_ (h)	12.79 ± 0.73	20.59 ± 6.35**
*T*_max_ (h)	3.83 ± 1.10	2.83 ± 0.75*
CL/F (L/h/kg)	6.91 ± 1.65	3.46 ± 1.13*
*C*_max_ (μg/L)	210.33 ± 26.08	315.3 ± 24.35**

*p < 0.05, *p < 0.01, ***p < 0.001.

### Succinic acid improved the metabolic stability of irbesartan

In rat liver microsomes, the half-life of irbesartan was 23.32 ± 3.46 min, and the intrinsic clearance rate was obtained as 59.43 ± 6.12 μL/min/mg protein. In the presence of succinic acid, the half-life of irbesartan was prolonged to 27.35 ± 2.15 min with the intrinsic clearance rate of 50.68 ± 5.64 μL/min/mg protein, indicating the improving metabolic stability of irbesartan by succinic acid.

### Succinic acid inhibited the activity of CYP2C9

**Figure 2.**
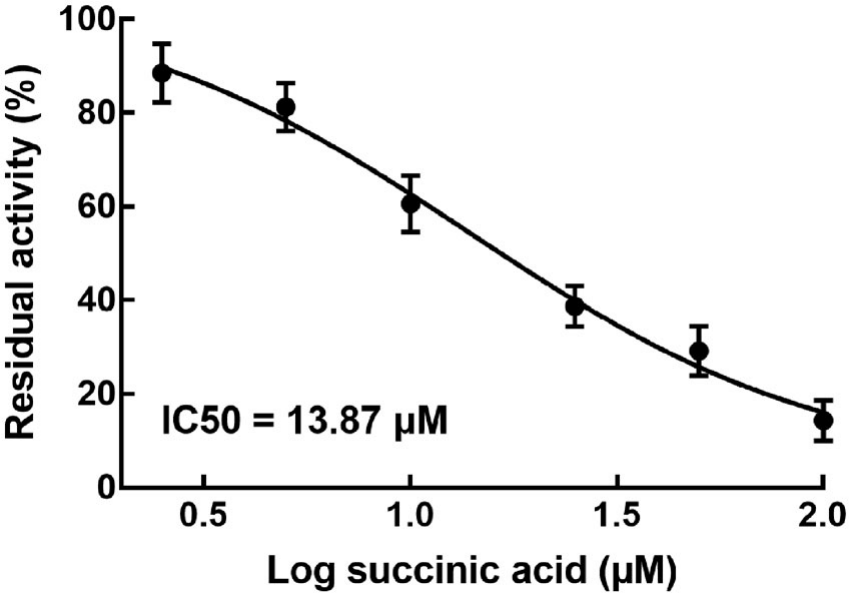
The effect of succinic acid (0, 2.5, 5, 10, 25, 50, and 100 μM) on the activity of CYP2C9 in rat liver microsomes.

The activity of CYP2C9 was found to decrease with the increase of succinic acid concentration. In the presence of various concentrations of succinic acid, succinic acid showed a dose-dependent manner in the inhibition of CYP2C9 with the IC_50_ values of 13.87 μM (Figure 2). The inhibition of CYP2C9 was speculated to be the reason for the increasing system exposure of irbesartan.

## Discussion

Adverse interactions between co-administrated are one of the major causes of therapy failure and even drug toxicity (Rekić et al. 2017; Amadi and Mgbahurike 2018). The activity of CYP450s has been considered as one of the mechanisms underlying the drug-drug interaction. In previous studies, this kind of interaction during co-administration of various drugs has been widely reported. For instance, verapamil is a specific inhibitor of CYP3A4, and this inhibitory effect induces its interaction with oridonin that increases the plasma concentration and prolonged the half-life of oridonin (Liu et al. 2019). The metabolism of warfarin involves CYP2C9 and CYP3A4, its co-administration with andrographolide results in the increasing system exposure of warfarin through inhibiting the activity of CYP3A4 or CYP2C9 (Zhang et al. 2018). Therefore, the study on the interaction between co-administrated drugs is necessary to avoid treatment failure or toxicity due to drug-drug interaction.

Both irbesartan and succinic acid are commonly used for the treatment of cardiovascular disease, due to their similar pharmacological effects and indications (Markham et al. 2000; Zhang et al. 2014). The interaction between these two drugs is critical to guide the clinical application of the combination of succinic acid and irbesartan. Here, the co-administration of succinic acid and irbesartan was investigated and their interaction was found. Succinic acid increased the AUC of irbesartan and prolong the half-life of irbesartan. The consistent *in vitro* results were also obtained that succinic acid enhanced the metabolic stability in rat liver microsomes. The increasing system exposure of irbesartan in the presence of succinic acid implies the potential side effects resulting from the high plasma concentration of irbesartan (Malishevskii 2012).

Additionally, the effect of succinic acid on the activity of CYP2C9 was estimated in rat liver microsomes. It was found that succinic acid significantly inhibited the activity of CYP2C9 in a dose-dependent manner, which is consistent with the previous study (Wang et al. 2020). Meanwhile, in the previous study, CYP2C9 has been demonstrated to participate in the metabolism of irbesartan (Bourrie et al. 1999). Hence, the inhibition of CYP2C9 was speculated to be the main reason for the interaction between succinic acid and irbesartan. However, despite the co-administrated drugs or herbs, the activity of CYP2C9 could be affected by many other environmental factors, such as genetic polymorphisms, smoking, and diet (Magliocco et al. 2019; Fabbri and Serretti 2020), which should be considered in the clinical prescription of co-administrated drugs or herbs.

From the above experimental findings, it was concluded that co-administration of succinic acid and irbesartan induced drug-drug interaction, which increased the plasma concentration and inhibited the metabolism of irbesartan through inhibiting the activity of CYP2C9. Therefore, the dose of irbesartan should be adjusted when co-administrated with succinic acid in the clinic.
